# Integration of single-cell regulon atlas and bulk RNA-seq for individualized prognostic prediction in stomach adenocarcinoma

**DOI:** 10.1016/j.isci.2026.116100

**Published:** 2026-05-22

**Authors:** Aiping Zhang, Zhe Li, Xin Jiang, Yaoyao Li, Min Zhang, Yanbing Ding

**Affiliations:** 1 Department of Gastroenterology, The Affiliated Hospital of Yangzhou University, Yangzhou University, Yangzhou, Jiangsu, China; 2 Department of Epidemiology, Center for Global Health, School of Public Health, Nanjing Medical University, Nanjing, Jiangsu, China

**Keywords:** Health sciences, Medicine, Oncology

## Abstract

Transcriptional regulators reflect cellular heterogeneity and are key for prognostic modeling. Given the poor prognosis of stomach adenocarcinoma (STAD), regulator-derived signatures are vital for risk stratification. Using multi-stage scRNA-seq data, we delineated the transcriptional regulatory landscape of STAD and identified *Helicobacter pylori*-associated epithelial heterogeneity in intestinal metaplasia. We then developed a 23-regulator machine learning-based STAD prognostic signature (SPS) from malignant epithelial cells to predict overall survival (OS). Patients with high-SPS exhibited significantly worse OS than patients with low-SPS (hazard ratio [HR] = 1.50, 95% CI: 1.09–2.09, log rank *p* = 7.11 × 10^−3^). Notably, SPS outperformed other established STAD prognosis models across various independent datasets and remained an independent prognostic factor after adjusting for clinical and pathologic factors. Moreover, integrating SPS with tumor stage and age showed superior accuracy to stage alone. Collectively, our study establishes a robust regulator-based prognostic signature, holding potential to facilitate precision prognostication in STAD.

## Highlights

• Single-cell transcriptional regulatory landscape in STAD
• Malignant epithelial cell-specific regulators-derived SPS predicts STAD prognosis
• SPS shows high accuracy and robustness, serves as an independent predictor for STAD

## Graphical abstract

**Figure:**
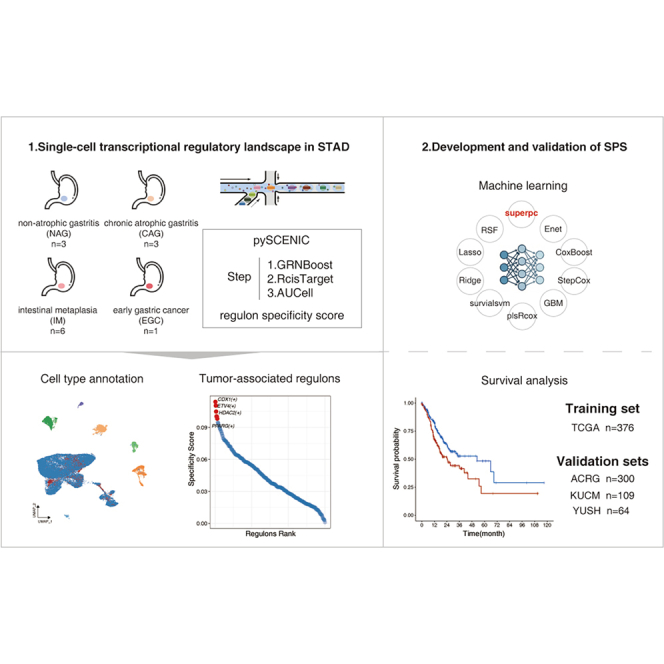

## Introduction

Gastric cancer (GC) has become the fifth most common cause of cancer incidence and mortality worldwide.^1^ The tumor’s aggressive progression and the common delay in diagnosis are major contributors to its high case-fatality rate.^2^ Despite the improved early diagnosis and treatment levels facilitated by the application of the endoscopic examination, as well as advancements in surgical methods and systemic therapies, the five-year survival rate for patients with advanced GC was still dismal.^3^ Consequently, the development of robust prognostic signatures is critical for guiding the individualized clinical management of stomach adenocarcinoma (STAD).

Transcriptional regulation plays a central role in controlling gene expression programs underlying both physiological and pathological processes. Dysregulation of transcriptional regulators has been shown to profoundly influence tumor progression, including the remodeling of the tumor microenvironment and adaptive resistance to therapy.^4^^,^^5^ Coordinated activity of regulatory factors and their associated gene expression programs defines cellular phenotypes, forming complex transcriptional networks commonly referred to as gene regulatory networks (GRNs) or regulons.^6^ Disruptions in GRNs can lead to aberrant expression of the associated genes and contribute to the development of diseases, particularly cancers.^7^

Single-cell RNA sequencing (scRNA-seq) technology enables the analysis of gene expression at the cellular level, thereby facilitating the investigation of tumor heterogeneity and the tracking of disease progression.^8^ Recently, scRNA-seq technology has been leveraged to infer transcriptional regulatory networks, allowing the characterization of transcriptional programs underlying diverse cell states within complex tissues.^9^ Accordingly, characterizing cell-type-specific transcriptional regulatory networks provides a framework for dissecting transcriptional heterogeneity in GC.

In this study, we inferred transcriptional regulatory features in GC using scRNA-seq. We further identified regulatory features specific to malignant gastric epithelial cells and developed a prognostic risk model for patients with STAD. This work provides additional insights into tumor heterogeneity and supports improved prognostic assessment and personalized patient management.

## Results

### Construction of a single-cell-specific transcriptional regulatory network in STAD

**Figure 1:**
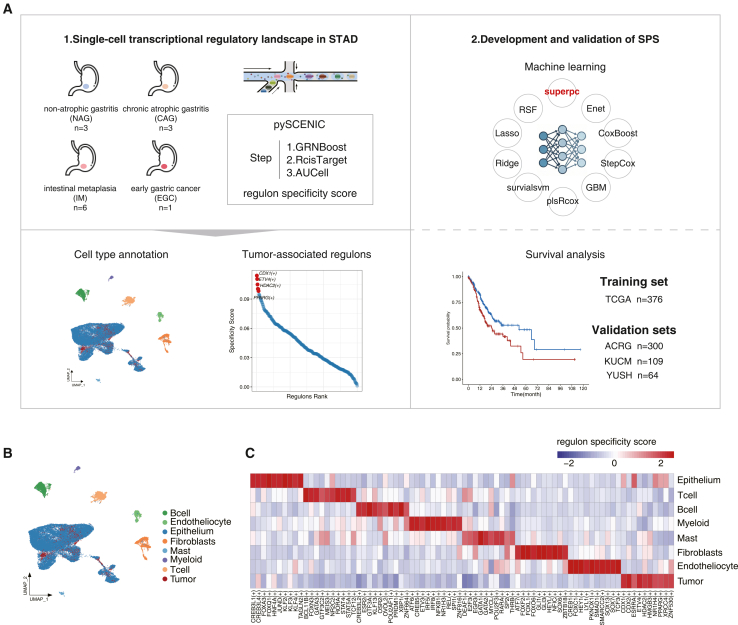
SCENIC analysis of human stomach adenocarcinoma single cell dataset (A) The workflow of this study. (B) The UMAP plot of 38,525 cells to visualize clusters colored by cell type. (C) The heatmap demonstrates specific regulons across cell types. The transcription factor (TF) regulatory activity (AUCell score) was estimated by using pySCENIC. The color intensity was proportional to the scaled AUCell score for each cell type, and the top 10 regulons of each cell type were shown.

A graphical summary of the research methodology is illustrated in Figure 1A. We mapped a single-cell transcriptional regulatory landscape in STAD using an scRNA dataset (GSE134520). This dataset includes a total of 38,525 cells from 13 samples representing the progressive histologic stages, i.e., 3 cases of non-atrophic gastritis (NAG), 3 cases of chronic atrophic gastritis (CAG), 6 cases of intestinal metaplasia (IM), and 1 case of early gastric cancer (EGC) (Figure 1A). The resulting 8 cell clusters were divided into 3 major cell lineages, including epithelial cells (*n* = 30,687), stromal cells (*n* = 2,690), and immune cells (*n* = 5,148) (Figure 1B). Subsequently, we employed the SCENIC algorithm to infer regulons for each cell subpopulation. By identifying co-expression modules between transcriptional regulators and candidate target genes, the algorithm constructs the underlying regulatory circuitry, defined as regulons (Figure 1C). We observed that some regulons were shared across cell types. Notably, many cell subpopulations displayed subset-specific regulons. For instance, findings consistent with ours demonstrate that *FOXQ1* mRNA is localized to the gastric epithelium.^10^ Meanwhile, *ESR2* exhibits marked expression in B cells.^11^ *POU2AF1* serves as a pivotal transcriptional coactivator for the POU2 family, modulating B cell development and malignant progression in B cells.^12^ Our results indicate that regulons could capture the heterogeneous GRNs underlying cellular diversity in STAD.

### Potential regulatory patterns in gastric epithelial cells associated with *Helicobacter pylori* infection

**Figure 2:**
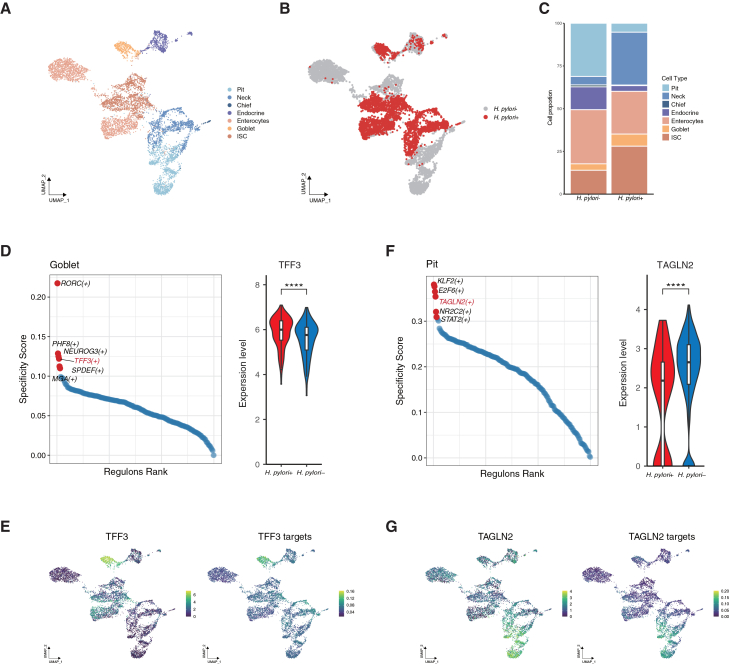
Epithelial-specific gene regulatory network in intestinal metaplasia (A) UMAP plot of the major clusters of epithelial cells from 6 IM samples. (B) UMAP plot of the epithelial cells from 3 *H. pylori*-infected IM samples and 3 uninfected IM samples. (C) The proportion of epithelial cell types, with or without *H. pylori* infection. (D and F) Left: Rank for regulons in goblet (D) and pit (F) cells based on the RSS. RSS, regulon specificity score. Right: Violin plots display the distribution of expression of *TFF3* (D) and *TAGLN2* (F), with or without *H. pylori* infection. Statistical significance was determined by the Wilcoxon rank-sum test. ∗∗∗∗*p* < 0.0001. (E and G) UMAP plots of the expression levels of transcriptional factor *TFF3* (left, E) and *TAGLN2* (left, G) and their target genes (right) in epithelial cells.

*Helicobacter pylori* colonizes the gastric epithelium and is a major risk factor for GC and precancerous lesions.^13^^,^^14^^,^^15^ Given its impact on gastric epithelial cells, we investigated changes in transcriptional regulatory features of gastric epithelial cells associated with *H. pylori* infection. Based on *H. pylori* infection status from 6 IM samples, we categorized epithelial cells into *H. pylori*-positive and *H. pylori*-negative groups (Figures 2A and 2B). We observed apparent differences in gastric epithelial cell composition and global gene expression patterns between *H. pylori*-positive and *H. pylori*-negative samples (Figures 2C and S1, and Table S1). In particular, neck cells (4.98%–30.93%), goblet cells (3.77%–7.11%), and ISC cells (13.95%–28.03%) appeared more frequent in the *H. pylori*-infected biopsies, whereas chief cells (1.12%–0.34%) and pit cells (31.13%–5.25%) appeared less frequent (Figure 2C). We subsequently focused on the cell type-specific regulons (Figure S2). Specifically, the *TFF3*-associated regulon exhibited elevated regulatory activity in goblet cells in *H. pylori*-infected biopsies (Figure 2D, 2E, and S1). Genes within this regulon, including *THBS2*, which has been previously linked to *H. pylori*-associated GC development and metastasis,^16^ showed goblet cell-enriched expression patterns (Figure 2E). Of note, among these regulon-associated genes, *FAM3D* and *ZG16B* displayed higher expression levels in *H. pylori*-infected samples (Figure S1). Meanwhile, the *TAGLN2*-associated regulon showed lower regulatory activity in pit cells (Figure 2F, 2G, and S1). To a certain extent, our study suggested an association between *H. pylori* infection and differences in cell type-specific regulatory states within gastric epithelial cells.

Table S1. Differential genes expressed in epithelial cells between *H. pylori*+ and *H. pylori*-tissues (|FoldChange|> 1.5, P < 0.05), related to Figure 2

### Development and validation of the STAD prognostic signature (SPS)

Moreover, we identified 175 transcriptional regulators that were preferentially associated with malignant epithelial cells. For example, *ETV4*, *ESRRA,* and other transcriptional regulators are significantly activated in malignant epithelial cells. Their overexpression in epithelial cells is implicated in the malignant progression of tumors (Figure 1C).^17^^,^^18^ Additionally, Gene Ontology (GO) enrichment analysis revealed that these transcriptional regulators were significantly enriched in gene sets related to transcription factor activation/inhibition (Figure S3A). We also used the MSigDB Hallmark gene sets and found the TNFA signaling via the NFKB gene set to be most significantly affected (Figure S3B).

**Figure 3:**
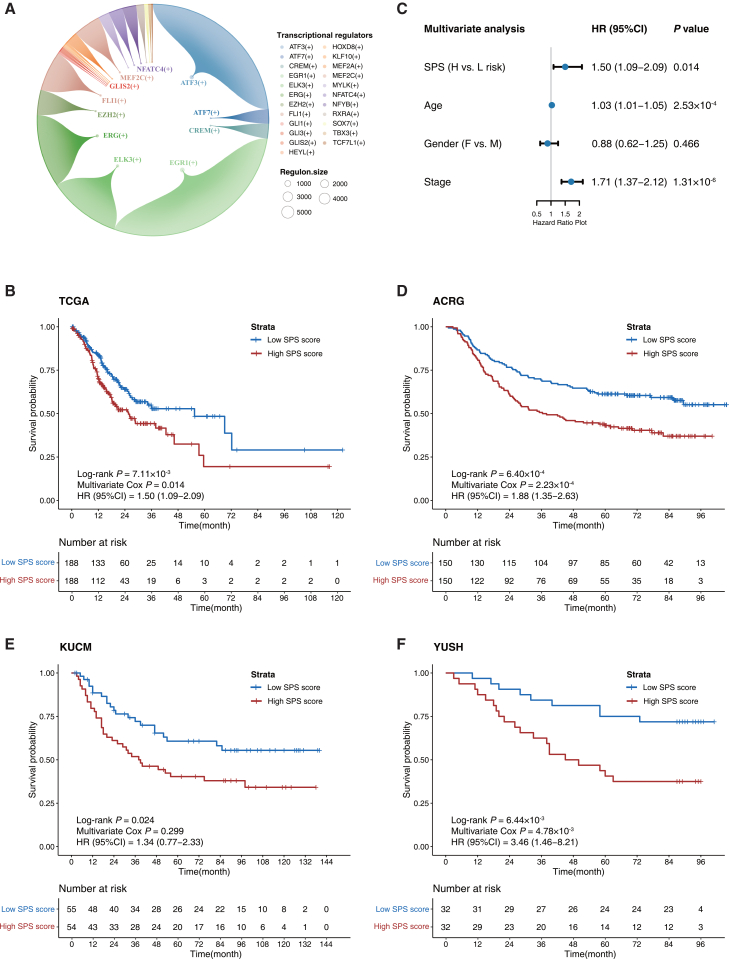
Development and validation of the SPS (A) Identification of prognostic regulons. Large bubbles represent the transcriptional regulators, while the peripheral areas indicate their targets. Bubble size is proportional to the number of target genes within each regulon. (B) Kaplan-Meier (KM) curve of the overall survival (OS) for patients between high- and low-risk groups in the TCGA discovery dataset. (C) Multivariate analysis results assessing the prognostic efficiency of SPS. Solid dots represent the HR, and the closed-ended horizontal lines represent the 95% CIs. (D–F) Kaplan-Meier (KM) curves of OS for patients between high- and low-risk groups in three validation datasets: ACRG (D), KUCM (E), and YUSH (F). CI, confidence interval; HR, hazard ratio.

Performing univariate Cox regression analysis on the 175 transcriptional regulators, setting *p* < 0.05 as the significance threshold, we identified 23 of them that were significantly associated with the prognosis of patients with GC (Figure 3A). Based on these 23 malignant epithelial-specific transcriptional regulators, we developed a prognostic signature for patients with STAD, defined as the STAD prognostic signature (SPS), using a machine learning-based integrative approach (Figure S4A). The TCGA dataset was designated as the training set, whereas the ACRG, KUCM, and YUSH datasets were employed as validation sets. By evaluating 117 combinations of 10 machine learning algorithms, we ultimately selected SuperPC to balance model complexity and predictive accuracy. This model achieved a mean C-index of 0.61 across all cohorts. The C-indices for the test cohorts ACRG, KUCM, and YUSH were 0.60, 0.62, and 0.64, respectively, all exceeding that of the discovery cohort (0.58), indicating robust performance (Figure S4B). By combining the risk score with the follow-up data, we stratified patients into high-risk or low-risk groups using the median value as the cutoff. Among the 376 patients with STAD with complete survival data in the TCGA, 188 were predicted to be high-risk, and 188 were predicted to be low-risk. The high-risk group demonstrated significantly worse overall survival (OS) than the low-risk group (Figure 3B, 5-year survival rate 19.5% vs. 48.4%, HR = 1.50, 95% CI: 1.09–2.09, log rank *p* = 7.11 × 10^−3^). To verify the robustness of the prognostic power of SPS, we employed a multivariate Cox regression model. Even after further adjusting for clinical and pathological factors such as tumor stage, gender, and age, the prognostic signature retained its significance as an independent prognostic factor (*p* < 0.05, Figure 3C). Then, we validated the prognostic potency of SPS in 3 independent validation datasets. In the Asian Cancer Research Group (ACRG) cohort with 300 patients with STAD, individuals in the high-risk group exhibited significantly worse OS compared to those in the low-risk group (Figure 3D, 5-year survival rate 43.2% vs. 61.3%, HR = 1.88, 95% CI: 1.35–2.63, log rank *p* = 6.40 × 10^−4^). Similar results were observed in the Kosin University College of Medicine (KUCM) cohort with 109 patients (Figure 3E, 5-year survival rate 40.3% vs. 60.7%, HR = 1.34, 95% CI: 0.77–2.33, log rank *p* = 0.024) and in the Yonsei University Severance Hospital (YUSH) cohort with 64 patients (Figure 3F, 5-year survival rate 40.6% vs. 75.0%, HR = 3.46, 95% CI: 1.46–8.21, log rank *p* = 6.44 × 10^−3^). Similarly, multivariate analysis with adjustment for clinical and pathologic factors in validation cohorts further confirmed the independent prognostic value of SPS (Figure S5). Furthermore, across both the training and validation cohorts, the SPS_stage yielded a modest but consistent improvement in predictive performance compared to the robust clinical staging system. This enhancement was evidenced by an incremental gain in the C-index (mean Δ_C-index_ was 0.017) (Table S2), as well as relative advantages observed in time-dependent ROC (Figure S6), calibration (Figure S7), and decision curve analysis (DCA) (Figure S8), driven primarily by improved long-term prognostic performance.

### Biological characteristics and preliminary validation of the SPS

We investigated the association between SPS and immune cell infiltration by CIBERSORTx and ssGSEA algorithms. As shown in Figure S9, we observed significant differences in immune cell infiltration patterns between high- and low- SPS groups. Specifically, the high SPS group exhibited reduced levels of immune-enhancing immune cells, including activated CD4^+^ T cells, and significantly elevated levels of immune-suppressive immune cells, such as mast cells, macrophages, and Treg cells. In addition, significant differences in eight cancer hallmark features were identified between the high- and low-SPS groups, with the high group showing higher activity (Figure S10A). Notably, the activities of the 23 SPS regulators were significantly correlated with these cancer hallmark pathways (Figure S10B).

To further substantiate the reliability of the SPS, we investigated the role of *GLIS2*, a key transcriptional regulator in our model. First, *GLIS2* exhibited significantly higher expression in GC samples compared to normal samples in both TCGA and GSE54129 cohorts (Figure S11A). We then silenced *GLIS2* expression in AGS GC cells using siRNAs targeting *GLIS2*. We performed RT-qPCR to confirm the knockdown efficiency at the mRNA level (Figure S11B). Further cell proliferation assays, including CCK-8 and EdU assays, demonstrated that the knockdown of *GLIS2* significantly inhibited cell proliferation (Figures S11C and S11D). These functional findings suggest that *GLIS2* acts as an oncogene in GC progression. Moreover, we investigated the downstream target genes of *GLIS2*. Based on SCENIC output and predictions from other databases (e.g., GTRD and ChIP-Atlas), *SYNPO* and *SH2B3* were suggested to be downstream targets of *GLIS2*. Consistent with this, we found a significant positive correlation between the expression of these genes and *GLIS2* (Figures S11E and S11F). Furthermore, RT-qPCR results showed that the expression levels of *SYNPO* and *SH2B3* were downregulated following *GLIS2* silencing (Figures S11E and S11F).

### Comparison of the predictive performance of SPS with other prognostic signatures

We compared the predictive performance of SPS with 3 previously published prognostic signatures, including 6-gene signature,^19^ 5-gene signature,^20^ and GPSGC.^21^ The results were summarized in Table 1 and Figure S5. Although the other 3 signatures exhibited distinct predictive performance for the OS across several datasets, the predictive effect of SPS was robust across all datasets, even after adjusting for other clinical and pathological factors.

**Table 1:** Survival analyses of existing prognostic signatures in four datasets

Datasets	Signatures	Log rank *P*	C-index^a^	HR^b^	Cox *P*^b^
TCGA	SPS	7.11 × 10^−3^	0.577	1.50	0.014
	6-gene signature	0.954	0.526	0.93	0.661
	5-gene signature	0.059	0.565	1.47	0.021
	GPSGC	0.012	0.599	1.62	0.004
ACRG	SPS	6.40 × 10^−4^	0.603	1.88	2.23 × 10^−4^
	6-gene signature	0.319	0.524	0.92	0.595
	5-gene signature	1.02 × 10^−6^	0.647	1.97	9.77 × 10^−5^
	GPSGC	1.58 × 10^−3^	0.622	1.50	0.016
KUCM	SPS	0.024	0.614	1.34	0.299
	6-gene signature	0.197	0.599	1.07	0.814
	5-gene signature	0.015	0.650	1.24	0.457
	GPSGC	0.103	0.629	1.10	0.728
YUSH	SPS	6.44 × 10^−3^	0.642	3.46	0.005
	6-gene signature	0.787	0.524	0.72	0.398
	5-gene signature	1.46 × 10^−3^	0.733	4.44	9.90 × 10^−4^
	GPSGC	0.541	0.599	1.59	0.220

C-index^a^ were generated by the univariate Cox proportional hazards model, HR^b^ and Cox *P*^b^ were generated by the multivariate Cox proportional hazards model.

### Integrated prognostic signature by combining the SPS with clinical factors

**Figure 4:**
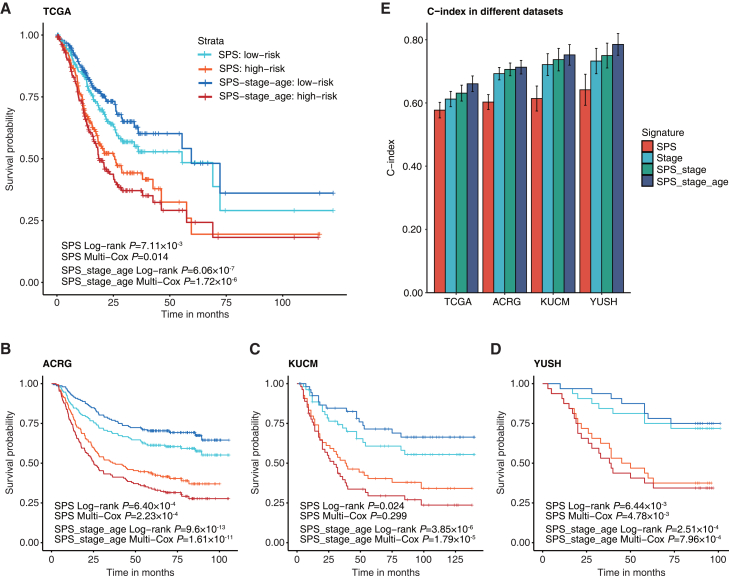
Performance of SPS_stage_age in discovery and validation datasets (A–D) The KM curves of OS for patients between high- and low-risk groups determined by SPS and SPS_stage_age signatures in the TCGA discovery dataset (A), and independent validation datasets ACRG (B), KUCM (C), and YUSH (D). (E) Comparison of the SPS_stage_age signature with SPS and tumor stage. Error bars in the bar graph represent the standard deviation of the C-index.

In the multivariate Cox regression analyses, the tumor stage, age, and SPS were identified as independent prognostic factors in all datasets, indicating their complementary value (Figure S5). To further improve the predictive performance, we derived a new signature in the TCGA dataset by combining tumor stage, age, and SPS, which was calculated as (0.41 × SPS) + (0.53 × stage) + (0.03 × age) and named as SPS_stage_age. In both discovery and validation datasets, we observed that the SPS_stage_age composite index yielded more pronounced prognostic distinctions compared to the binary SPS alone (Figures 4A–4D). Compared to individual factors such as tumor stage, or SPS alone, the integrated SPS_stage_age index exhibited enhanced accuracy in predicting survival across all datasets (Figure 4E).

## Discussion

Characterizing GRNs is essential in cancer research for elucidating both cancer prognosis and gene interactions.^22^ In this study, we employed scRNA-seq data from GSE134520 to construct a single-cell regulatory network atlas for STAD using the SCENIC algorithm and explored differences associated with *H. pylori* infection status within this framework. We further developed a novel prognostic risk model termed the SPS and validated it in both training and validation datasets. Notably, this model enhanced the accuracy of OS estimation in STAD by integrating molecular and clinical features, thereby supporting more informed clinical decision-making.

In our study, we noted heterogeneity in cell-type-specific transcriptional regulation within the gastric epithelium associated with *H. pylori* infection. Notably, the inferred regulatory differences appear to be cell-type specific, with distinct regulatory programs observed across goblet cells, pit cells, and other epithelial lineages. Within this framework, the *TFF3*- and *TAGLN2*-associated regulons served as representative examples of infection-associated regulatory state shifts in different epithelial lineages, consistent with previous studies.^23^^,^^24^ While our analyses are inferential and do not establish direct mechanistic regulation, these observations provide insights into the regulatory patterns associated with *H. pylori* infection.

In addition, we established the SPS, a scoring index comprising 23 transcriptional regulators with prognostic significance. To explore the biological significance of this transcriptional regulatory signature, we examined the core constituent genes. Notably, several candidate transcriptional regulators have been implicated in GC tumorigenesis. For instance, existing studies indicate that *EGR1* promotes GC progression and metastasis by activating various downstream genes through distinct mechanisms, such as promoting cell cycle progression or reprogramming fatty acid metabolism.^25^^,^^26^ The E26 transformation-specific (ETS) transcription factor *ELK3* is required for the migration and invasion of GC cells.^27^ *GLI1* acts as a direct transcriptional target and a reliable indicator of Hedgehog (Hh) signaling pathway,^28^ a signaling cascade that is critical for tumorigenesis and sustaining cancer stemness. Previous research has indicated that *GLI1* can be activated by the AKT-mTOR pathway. The co-expression of *GLI1* and *p*-AKT is related with aggressive cellular functions such as proliferation, migration, and drug-resistance, and could serve as an indicator of poor prognosis in GC.^29^ A study investigating the genetic susceptibility to GC revealed that *GLI3* promotes GC progression and correlates with adverse outcomes in patients.^30^ Findings from these molecular mechanism studies could provide evidence for the reliability of our prognostic model.

In the TCGA training dataset, the SPS reveals that the five-year survival rate in the high-risk group was significantly lower than that of the low-risk group, and this result was consistent in the validation sets. Importantly, compared to the risk models proposed by Cho et al. (the 6-gene signature),^19^ Zhang et al. (the 5-gene signature),^20^ and Cai et al. (gene expression-based prognostic signature (GPSGC)),^21^ our risk score (SPS) demonstrates superior accuracy in predicting patient survival. This highlights the robust predictive power of SPS for patient outcomes. Moreover, our analysis revealed that prognostic models integrating gene expression profiling demonstrated better predictive accuracy than the conventional clinical model utilizing only tumor stage information. By constructing malignant epithelial cell-specific transcriptional regulatory networks, we uncovered the regulatory essence of tumor progression and its association with clinical outcomes, while also eliminating the influence of tumor heterogeneity and varying tumor cell proportions.

In conclusion, our findings depict a comprehensive landscape of the single-cell regulon atlas, uncovering the potential heterogeneity of GRNs within STAD tissues, and offer preliminary insights into *H. pylori*-associated epithelial alterations. We further developed an individualized prognostic signature using the malignant epithelial-specific transcriptional regulators for the survival stratification of patients with STAD. This signature holds promise as a valuable predictive tool for STAD survival, suggesting potential for further personalized clinical management of patients with STAD. Its ability to potentially inform treatment decisions highlights its translational significance.

### Limitations of the study

Although we constructed a robust signature and validated its predictive efficiency, this study has several limitations. Firstly, refining a well-established prognostic standard such as the clinical stage is inherently challenging; however, the modest yet steady gains provided by the SPS_stage represent a meaningful marginal improvement in risk stratification. The sustained performance across multiple cohorts and metrics validates the potential of the SPS_stage as a reliable supplementary tool to enhance conventional staging, arguing against overfitting. Nevertheless, the prognostic efficacy of SPS was tested in retrospective cohorts, and its clinical utility requires independent validation in larger, prospective cohorts of patients with STAD. Additionally, the generalizability of the findings might be constrained by the sample size in this study, highlighting the need for future research involving larger-scale validation to confirm the model’s robustness. Secondly, patient prognosis may also be influenced by other clinicopathological variables, such as treatment regimens, tumor burden, and comorbidities. Unfortunately, similar to most large-scale databases, detailed information on these factors is unavailable in the datasets used in our study. Further research incorporating these variables is warranted to validate and expand upon our findings. Thirdly, regarding regulon inference, while SCENIC is a powerful tool, the regulators it identifies remain computational predictions. To bolster the reliability of our inferences, we provided supporting evidence by incorporating validation from multiple datasets as well as performing some functional experiments on the key regulator *GLIS2*. However, further experimental validation is warranted to confirm the underlying mechanisms in future studies. Regarding *H. pylori* infection-related stratification, we acknowledge the current absence of single-cell validation cohorts and the inherent challenges in performing functional validation at the single-cell level. Consequently, while our data highlight significant alterations in *TFF3* and *TAGLN2*, their clinical implications warrant cautious interpretation. To further solidify these insights, future efforts should prioritize epithelial cell subclassification through both advanced data analytics and organoid experiments. Fourthly, the immediate clinical application of the 23-gene signature may face challenges. However, this model represents an optimal trade-off between complexity and prognostic accuracy, as reducing the gene count would compromise performance. While current transcriptomic platforms make such panels increasingly feasible, cost and implementation remain critical barriers. Therefore, developing simpler, clinically tractable alternatives, such as IHC panels focusing on key regulators, is a crucial direction for future research to enhance translational potential. Finally, despite the fact that the scRNA-seq regulatory network prognostic model outperforms conventional transcriptomic features, the inherent limitations in information content and dimensionality may compromise the accuracy and comprehensiveness of inferred gene regulatory interactions compared to single-cell multi-omics. Specifically, during GC development, the transcriptional dysregulation of specific genes may also be influenced by epigenetic mechanisms such as histone modifications, DNA methylation, and non-coding RNAs.^31^

## Resource availability

### Lead contact

Requests for further information and resources should be directed to and will be fulfilled by the lead contact, Yanbing Ding (ybding@yzu.edu.cn).

### Materials availability

This study did not generate new unique reagents.

### Data and code availability

The datasets employed in this work can be accessed via the TCGA (https://portal.gdc.cancer.gov/) and GEO (https://www.ncbi.nlm.nih.gov/geo/). Detailed information about datasets is provided in Table S3. The code for our main analysis is available at https://github.com/AP-Zhang/Regulon-based_Survival_Pipeline. Further details and additional supporting data are available upon request from the corresponding authors.

## Acknowledgments

This research was supported by the 10.13039/501100001809National Natural Science Foundation of China (no. 82273084), the NSFC Young Scientists Fund (no. 82404358), the Postgraduate Research & Practice Innovation Program of Jiangsu Province (KYCX22_3569), and the Health Key Project of the Basic Research Program of Yangzhou (Joint Special Project, No. 2024-1-05).

## Author contributions

A.Z.: conceptualization, data curation, formal analysis, methodology, visualization, writing - original draft, and writing - review and editing. Z.L.: data curation, formal analysis, methodology, visualization, and writing - review and editing. X.J.: validation. Y.L.: funding acquisition and supervision. M.Z.: funding acquisition and supervision. Y.D.: funding acquisition, project administration, and supervision. All authors have read and approved the manuscript.

## Declaration of interests

The authors declare no competing interests.

## Declaration of generative AI and AI-assisted technologies in the writing process

During the preparation of this work, the authors used Zhipu Qingyan (powered by ChatGLM) in order to improve readability and language. After using this tool, the authors reviewed and edited the content as needed and take full responsibility for the content of the published article.

## STAR★Methods

### Key resources table

**Table:** 

REAGENT or RESOURCE	SOURCE	IDENTIFIER
**Chemicals, peptides, and recombinant proteins**		

RPMI 1640 medium	VivaCell	C3010-0500
Fetal Bovine Serum (FBS)	BioChannel	BC-SE-FBS01
Penicillin-Streptomycin	Gibco	15140122
jetPRIME	Polyplus	101000046
Cell Counting Kit-8 (CCK-8)	Dojindo	CK04-500T
EdU assay kit	RiboBio	C10310-1

**Critical commercial assays**		

TRIzol reagent	Invitrogen	15596018
PrimeScript RT reagent Kit with gDNA Eraser	TaKaRa	RR047A
TB Green Premix Ex Taq	TaKaRa	RR420A

**Deposited data**		

scRNA-seq data	GEO database	GSE134520
TCGA-STAD bulk RNA-seq data	NIH GDC data portal	https://portal.gdc.cancer.gov
microarray dataset (ACRG)	GEO database	GSE62254
microarray dataset (KUCM)	GEO database	GSE26901
microarray dataset (YUSH)	GEO database	GSE13861

**Experimental models: Cell lines**		

human gastric cancer cell line	IMMOCELL, Xiamen	AGS

**Oligonucleotides**		

See Tables S4 and S5	N/A	N/A

**Software and algorithms**		

R software	https://www.r-project.org	v4.3.3
inferCNV	https://github.com/broadinstitute/infercnv	v1.20.0
pySCENIC	https://github.com/aertslab/pySCENIC	v0.12.1
clusterProfiler	https://bioconductor.org/packages/release/bioc/html/clusterProfiler.html	v4.14.6
CIBERSORTx	https://cibersortx.stanford.edu	N/A
Survival	https://cran.r-project.org/web/packages/survival/index.html	v3.8.3
Survminer	https://cran.r-project.org/web/packages/survminer/index.html	v0.4.9
Seurat	https://cran.r-project.org/web/packages/Seurat/index.html	v4.4.0
GraphPad Prism	https://www.graphpad.com/	V10

### Experimental model and study participant details

#### Data collection

We analyzed gene expression profiles of 858 patients from 5 stomach adenocarcinoma (STAD) cohorts, including one scRNA-seq dataset, one bulk RNA-seq dataset and 3 microarray datasets, as detailed in Table S3. The STAD cancer scRNA-seq data (GSE134520)^32^ were downloaded from the Gene Expression Omnibus (GEO) (https://www.ncbi.nlm.nih.gov/geo/) database for analysis.

The bulk RNA-seq data and clinical information of 376 STAD patients were obtained from The Cancer Genome Atlas (TCGA, https://portal.gdc.cancer.gov). We retained genes with median TPM greater than 1. We also evaluated the predictive efficiency of our signature in three human GC microarray datasets (GSE62254 [Asian Cancer Research Group (ACRG) cohort], GSE26901 [Kosin University College of Medicine (KUCM) cohort]), and GSE13861 [Yonsei University Severance Hospital (YUSH) cohort] from the GEO database. Our study was restricted to STAD patients who had available transcriptomic data along with full clinical details, including age, gender, stage, and overall survival time. All gene expression values were log2-transformed (log2(x + 1)) within each dataset prior to survival analyses.

#### Cells

The AGS gastric cancer cell line (Sex: Female) was obtained from IMMOCELL (Xiamen, Fujian, China). AGS cells were cultured in RPMI 1640 medium containing 10% fetal bovine serum (FBS) and 1% antibiotics at 37°C in a humidified incubator of 5% CO_2_.

### Method details

#### Pre-processing of single-cell RNA sequencing data

The single-cell RNA sequencing dataset was processed using Seurat (v4.4.0).^33^ To remove low quality cells and likely multiple captures, we filtered out cells with the following criteria: UMI counts <500 or >20,000, gene counts <400 or >4,500, and mitochondrial content >20%. After filtering, we retained 38,525 cells for downstream analysis. Key QC metrics (UMI, nFeature, and mitochondrial content) for each sample were evaluated to ensure data quality. We applied the NormalizeData and ScaleData functions to normalize and scale the gene expression data. Subsequently, we identified the highly variable genes (HVGs) using FindVariableFeatures to perform principal component analysis. We selected the top 30 principal components and performed clustering using FindClusters. Batch effect correction was performed with Harmony,^34^ and dimensionality reduction was achieved via UMAP, yielding a two-dimensional embedding for visualizing cellular.

We then assigned cell type annotation to each cluster based on the expression of specific marker genes.^35^ Specifically, immunological cells were categorized into B cells (*CD79A* and *MZB1*), T cells (*CD3D* and *CD2*), mast cells (*CPA3* and *TPSB2*), and myeloid cells (*CSF1R* and *CD14*). Stromal cells were identified by the expression of *DCN* and *COL1A2*. Epithelial cells were also identified using a range of markers, including *EPCAM* and *KRT18*, and further subclassified into various types, including neck cells (*MUC6*), pit cells (*MUC5AC* and *TFF1*), chief cells (*PGA3* and *PGA4*), parietal cells (*ATP4A* and *ATP4B*), endocrine cells (*CHGA* and *CHGB*), enterocytes (*FABP1* and *APOA1*), goblet cells (*MUC2* and *ITLN1*) and intestinal stem cells (*OLFM4*).

#### Identification of malignant epithelium

In this study, we calculated the copy number variations (CNVs) score of each gastric cell using the “inferCNV” v1.20.0 R package. The inferCNV analysis was performed with default parameters. Epithelial cells from normal gastric samples served as the reference. We conducted hierarchical clustering on the CNV scores of all genes via the k-means algorithm. We identified non-malignant epithelial cells as those with low CNV scores and clustered with reference cells. Conversely, cells exhibiting elevated CNV levels were designated as malignant.

#### Transcriptional regulatory networks analysis

To identify transcriptional regulatory networks in STAD, SCENIC algorithm was implemented by pySCENIC (Single-Cell rEgulatory Network Inference and Clustering, v0.12.1).^36^ First, GENIE3 was employed to infer co-expression based regulatory relationships linking transcriptional regulators to candidate target genes. Motif enrichment analysis was then performed using RcisTarget to refine these relationships and identify putative direct regulatory interactions. Subsequently, regulon activity was quantified at the single-cell level using the AUCell algorithm, which calculates the Area Under the Curve (AUC) score for each regulon. The regulon specificity score (RSS) developed by Suo et al.^37^ was utilized to assess the cellular specificity of the inferred regulons.

#### Gene set enrichment analysis

The Gene Ontology (GO) enrichment analyses, encompassing Biological Process (BP), Molecular Function (MF), and Cellular Component (CC), as well as pathway analyses using HALLMARK gene sets from the Molecular Signatures Database (MSigDB), were conducted using the R package clusterProfiler (v4.14.6). An adjusted *p*-value cutoff of <0.05 was applied to determine significant enrichment of GO terms and HALLMARK pathways, with the top 10 of each visualized.

#### Construction of a gene signature from prognostic regulons

Among malignant epithelial-specific regulons, we identified transcriptional regulators that were significantly associated with patients’ overall survival (OS) by univariate cox regression analysis (*p* < 0.05). Subsequently, we performed an integrative machine learning analysis using the “mime” R package (v0.13)^38^ to construct a risk signature based on these cell-type specific transcriptional regulators, which we termed the STAD prognostic signature (SPS). The prognosis of patients was predicted in the training dataset (TCGA) and three validation datasets (ACRG, KUCM, and YUSH) by evaluating a total of 117 combinations of 10 machine learning algorithms incorporating these regulators. We further calculate Harrell’s concordance index (C-index) for each model on the TCGA, ACRG, KUCM, and YUSH datasets. The model exhibiting the highest mean C-index was identified as optimal. We then stratified the STAD cohort into SPS-high and SPS-low groups using the median score as the cutoff.

#### Evaluation of model predictive performance and clinical utility

The improvement in model fit and predictive accuracy was assessed by comparing the SPS_stage with the conventional clinical stage using C-index and time-dependent ROC analysis. Calibration curves were generated to assess the agreement between predicted survival probabilities and actual observations. Decision Curve Analysis (DCA) was employed to evaluate the clinical net benefit across a spectrum of threshold probabilities. All of these evaluations were performed in the training dataset (TCGA) and three validation datasets (ACRG, KUCM, and YUSH).

#### Assessment of immune cell infiltration and tumor-related features enrichment

To comprehensively compare the immune microenvironment and tumor-related pathway activities between SPS-high and SPS-low groups, we performed the following analyses. Immune cell infiltration was deconvoluted using CIBERSORTx (https://cibersortx.stanford.edu), an algorithm that estimates the relative abundances of 22 immune cell types via support vector regression. For mutual validation, immune cell proportions were also quantified by single-sample gene set enrichment analysis (ssGSEA).^39^ In parallel, ssGSEA was applied to evaluate the activity of tumor-related pathways, generating corresponding enrichment scores for each sample.^39^ Both the immune cell fractions and pathway enrichment scores were subsequently integrated for downstream comparative statistics and visualization.

#### Transfections

All control and target siRNAs were purchased from GenePharma (Shanghai, China). Transfection was performed using jetPRIME (Polyplus, France) according to the manufacturer’s protocol. At 48 h post-transfection, cells were collected for subsequent analysis. The siRNA sequences were provided in the Table S4.

#### Real-time quantitative PCR (RT-qPCR)

Total RNA was extracted using TRIzol Reagent (Invitrogen, USA) following the manufacturer’s instructions. The following cDNA was generated with PrimeScript RT reagent Kit with gDNA Eraser (TaKaRa, Japan). Subsequently, RT-qPCR was performed in duplicate using TB Green Premix Ex Taq (TaKaRa) on LightCycler 480 (Roche, Switzerland). GAPDH served as an internal control for normalization. Relative gene expression changes were calculated using the 2^-ΔΔCt^ method. The RT-qPCR primer sequences were listed in the Table S5.

#### Cell proliferation assays

Cell proliferation in our study was assessed using the Cell Counting Kit-8 (CCK-8, Dojindo, Japan) and the Cell-Light EdU Apollo In Vitro Kit (EdU, RiboBio, China) according to the standard protocol. For the CCK-8 assay, the Infinite M200pro microplate reader (Tecan, Switzerland) was employed to measure absorbance at 450 nm at 0, 1, 2, 3, and 4 days. For the EdU assay, the percentage of EdU-positive cells was quantified by high-content analysis on the ArrayScan VTI system (Thermo Fisher Scientific, USA).

### Quantification and statistical analysis

All statistical analyses in the study were performed using R software (v4.3.3; https://www.r-project.org/) and GraphPad Prism (v10). Survival analyses were conducted using the R packages “survival” (v3.8.3) and “survminer” (v0.4.9). Survival curves for overall survival (OS) of STAD patients were estimated using the Kaplan-Meier (KM) method, with statistical significance determined by the log rank test. The Cox proportional hazards regression model was utilized to assess the independent predictive value of the signature and estimate the hazards ratios (HRs) and 95% confidence intervals (95% CIs), after adjustment for age, gender, and tumor stage. All bootstrap validations were performed with 1,000 iterations to ensure model robustness. Correlations between continuous variables were evaluated using Spearman’s rank correlation. Group comparisons were performed using two-sided Wilcoxon rank-sum test. All *in vitro* experiments were conducted with at least three independent biological replicates. Data are represented as mean ± SD. The corresponding figure legends describe the statistical test for each experiment, the number of data points (n) analyzed, what n represents, and the number of experimental replicates performed. For single-variable comparisons across multiple groups (e.g., RT-qPCR analysis), one-way ANOVA followed by Dunnett’s post hoc test was employed. For CCK-8 proliferation assays, two-way ANOVA followed by Dunnett’s multiple comparisons test was used. Statistical significance was defined as *p* < 0.05. In the figures, significance is denoted by the following symbols: ∗*p* < 0.05, ∗∗*p* < 0.01, ∗∗∗*p* < 0.001, ∗∗∗∗*p* < 0.0001.
