# Supplementary material for: Integration of single-cell regulon atlas and bulk RNA-seq for individualized prognostic prediction in stomach adenocarcinoma
Source: iScience. 2026 May 22;29(6):116100. doi: 10.1016/j.isci.2026.116100 (PMC13217890; doi:10.1016/j.isci.2026.116100)
Supplement: Document S1. Figures S1–S11, and Tables S2–S5 [file mmc1.pdf]

iScience, Volume *29*

Supplemental information

Integration of single-cell regulon atlas and bulk RNA-seq for individualized prognostic prediction in stomach adenocarcinoma

Aiping Zhang, Zhe Li, Xin Jiang, Yaoyao Li, Min Zhang, and Yanbing Ding

Integration of single-cell regulon atlas and bulk RNA-seq for individualized prognostic prediction in stomach adenocarcinoma

Aiping Zhang, Zhe Li, Xin Jiang, Yaoyao Li, Min Zhang, Yanbing Ding

Figure S1. Volcano plot of differential gene expression analysis between *H. pylori* positive and negative epithelial cells.

Figure S2. SCENIC analysis of gastric intestinal metaplasia single cell dataset.

Figure S3. Gene set enrichment analysis of malignant epithelial-specific transcriptional regulators.

Figure S4. Development of the SPS.

Figure S5. The forest plots for overall survival (OS).

Figure S6. Time-dependent ROC curves of SPS_stage and stage only model for predicting overall survival (OS).

Figure S7. Development and validation of a composite nomogram integrating SPS and clinical stage for overall survival (OS) prediction in STAD.

Figure S8. Decision curve analysis (DCA) comparing the SPS_stage and Stage models for predicting 1-, 3-, and 5-year overall survival (OS) in STAD.

Figure S9. Differences in immune infiltration characteristics in the high- and low-SPS groups.

Figure S10. Functional characteristics of the SPS regarding cancer hallmarks.

Figure S11. *GLIS2* knockdown suppresses gastric cancer cell proliferation.

Table S2. Comparison of C-index values: SPS_ stage model vs. Stage model.

Table S3. Datasets used in this study.

Table S4. siRNA sequences.

Table S5. RT-qPCR primer sequences.

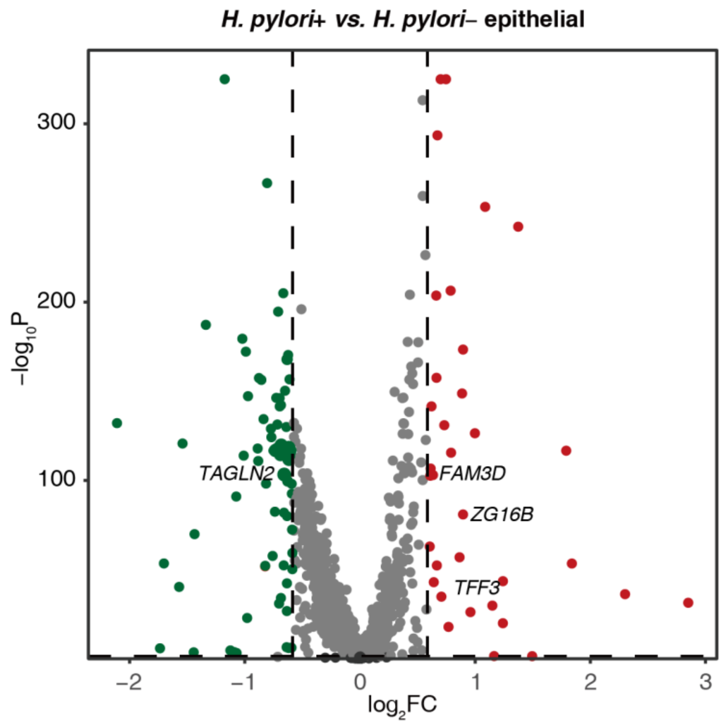

Figure S1. Volcano plot of differential gene expression analysis between *H. pylori* positive and negative epithelial cells.

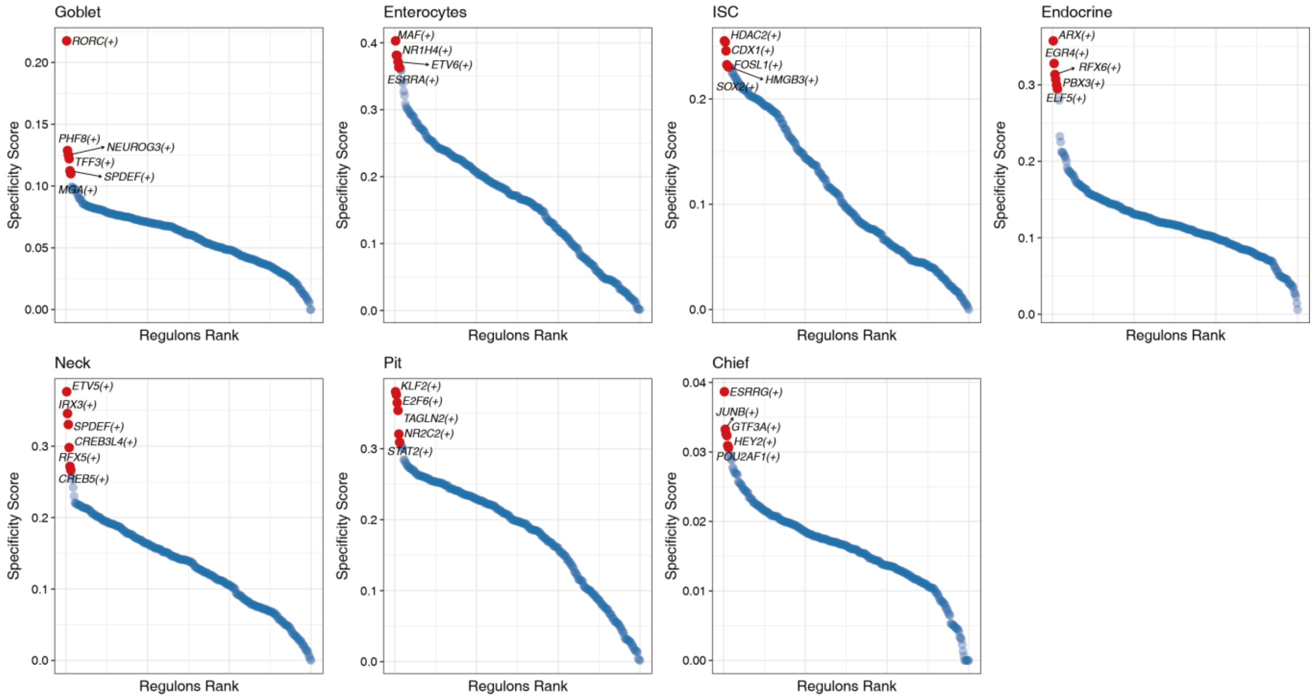

Figure S2. SCENIC analysis of gastric intestinal metaplasia single cell dataset.

SCENIC analysis predicting the potential transcriptional regulons across major clusters of epithelial cells from 6 IM samples.

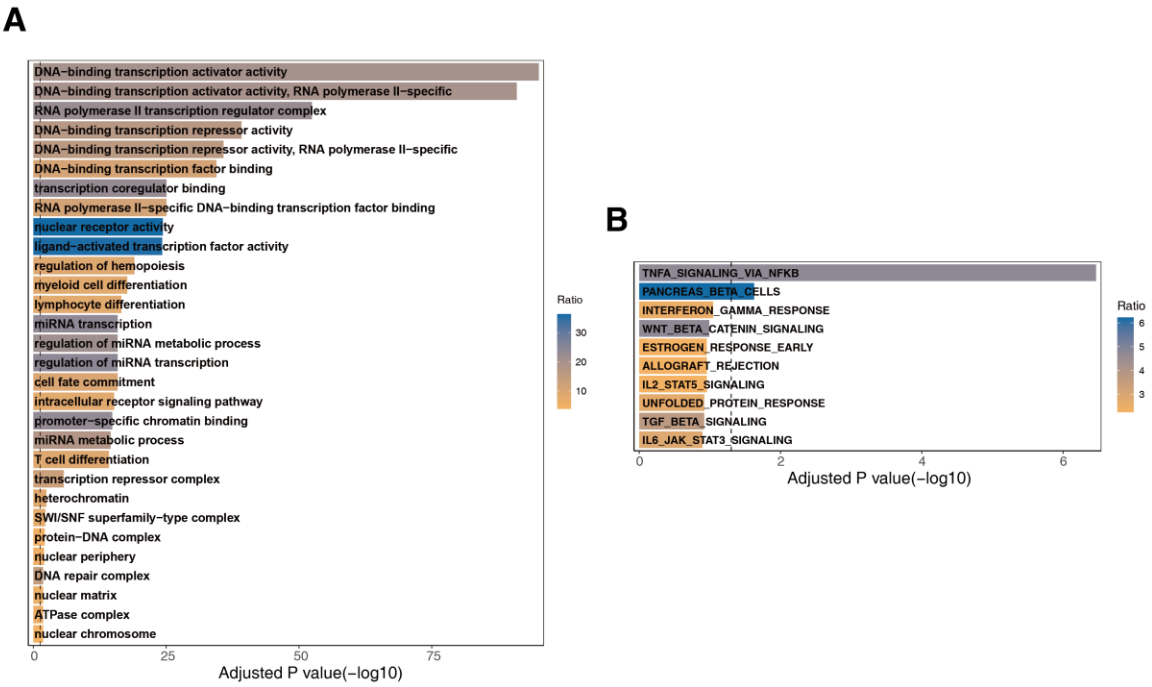

Figure S3. Gene set enrichment analysis of malignant epithelial-specific transcriptional regulators.

(A) Gene Ontology (GO) enrichment analysis of malignant epithelial-specific transcriptional regulators. **(B)** Pathway analyses of malignant epithelial-specific transcriptional regulators using HALLMARK gene sets from MSigDB.

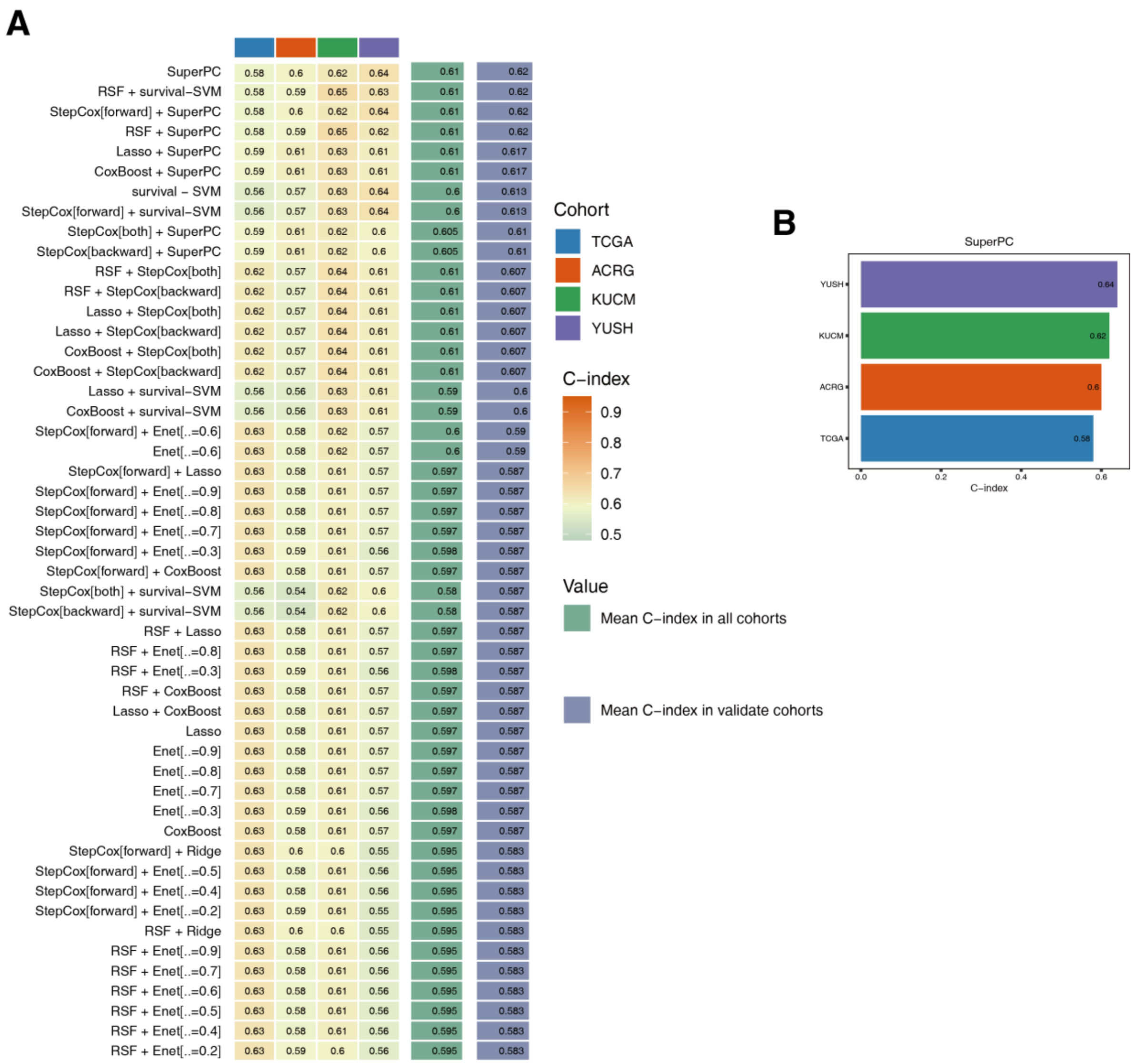

Figure S4. Development of the SPS.

(A) The top50 of 117 combinations of machine learning predictions with the highest average C-index in the four datasets were shown. **(B)** C-index of the model with the best predictive performance in the four datasets.

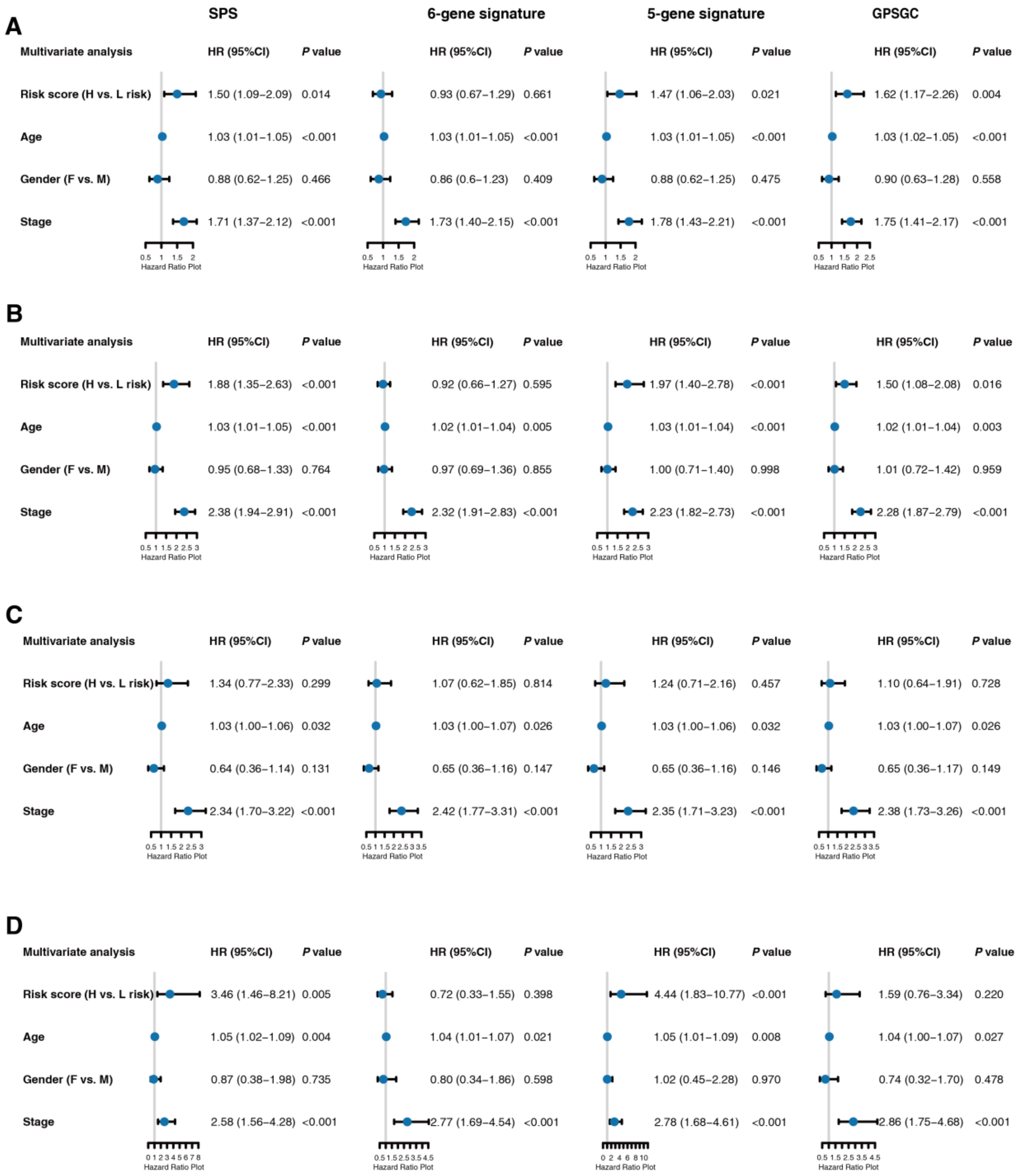

Figure S5. The forest plots for overall survival (OS).

The multivariate Cox regression analyses demonstrated the association between different prognostic signatures and OS in TCGA **(A)**, ACRG **(B)**, KUCM **(C)**, and YUSH **(D)** datasets.

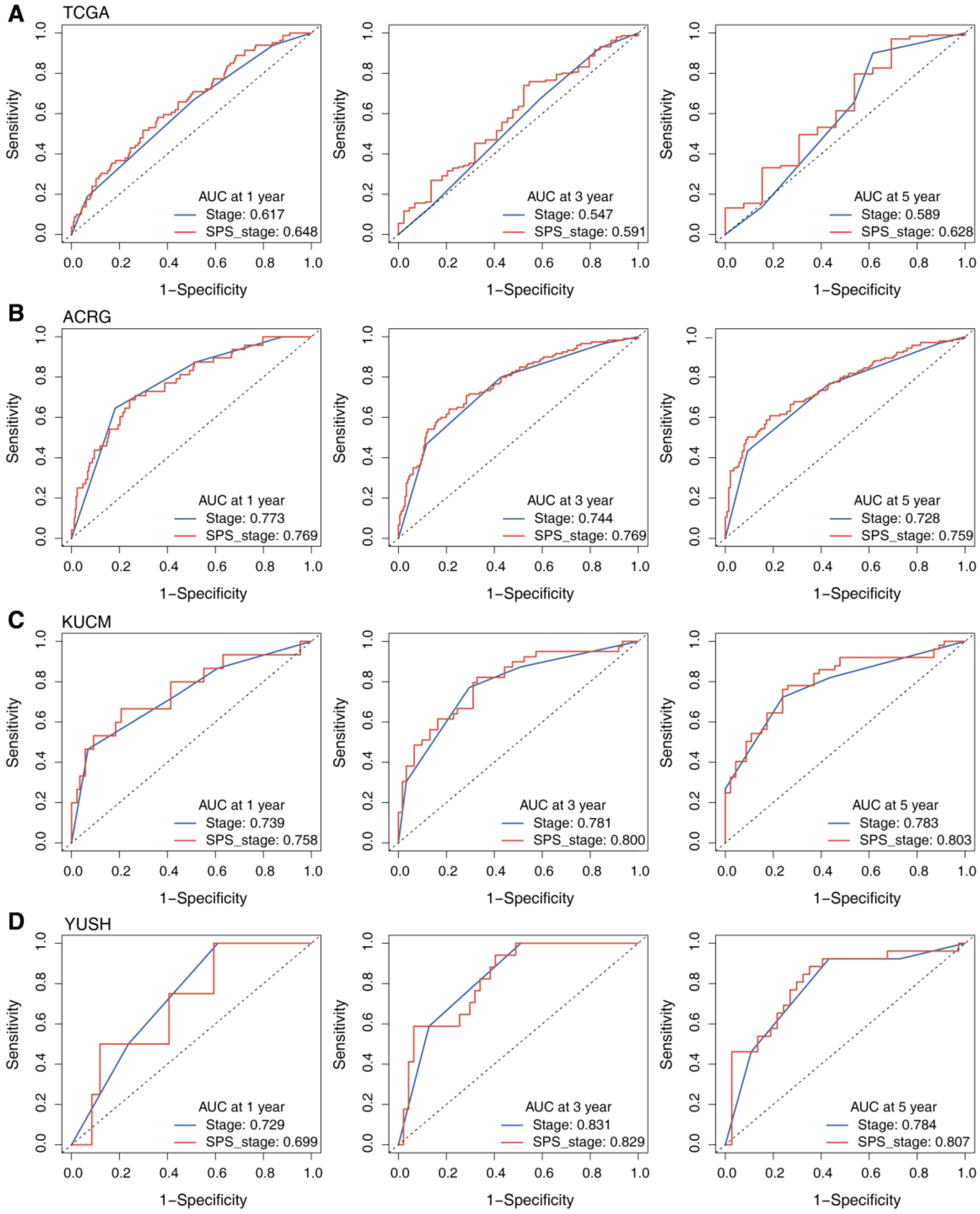

Figure S6. Time-dependent ROC curves of SPS_stage and stage only model for predicting overall survival (OS).

(A-D) Time-dependent ROC curves for SPS at 1-, 3-, and 5-years in **(A)** TCGA, **(B)** ACRG, **(C)** KUCM and **(D)** YUSH datasets. ROC, receiver operating characteristic. AUC, area under the curve.

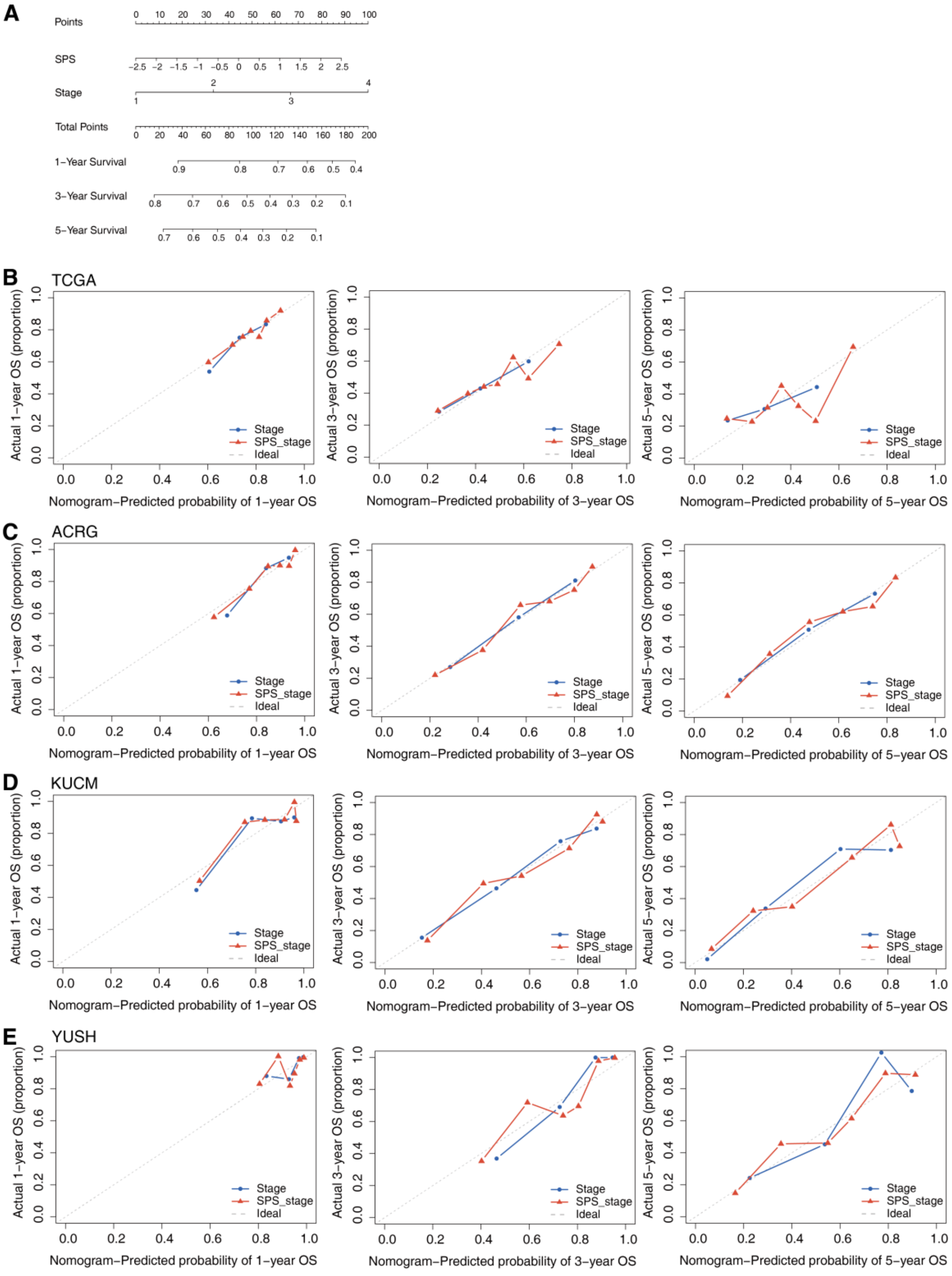

Figure S7. Development and validation of a composite nomogram integrating SPS and clinical stage for overall survival (OS) prediction in STAD.

(A) Nomogram including stage and SPS for personalized OS prediction. **(B-E)** Calibration plots of the nomogram to predict 1-, 3-, and 5-year OS in TCGA **(B)**, ACRG **(C)**, KUCM **(D)** and YUSH **(E)** datasets.

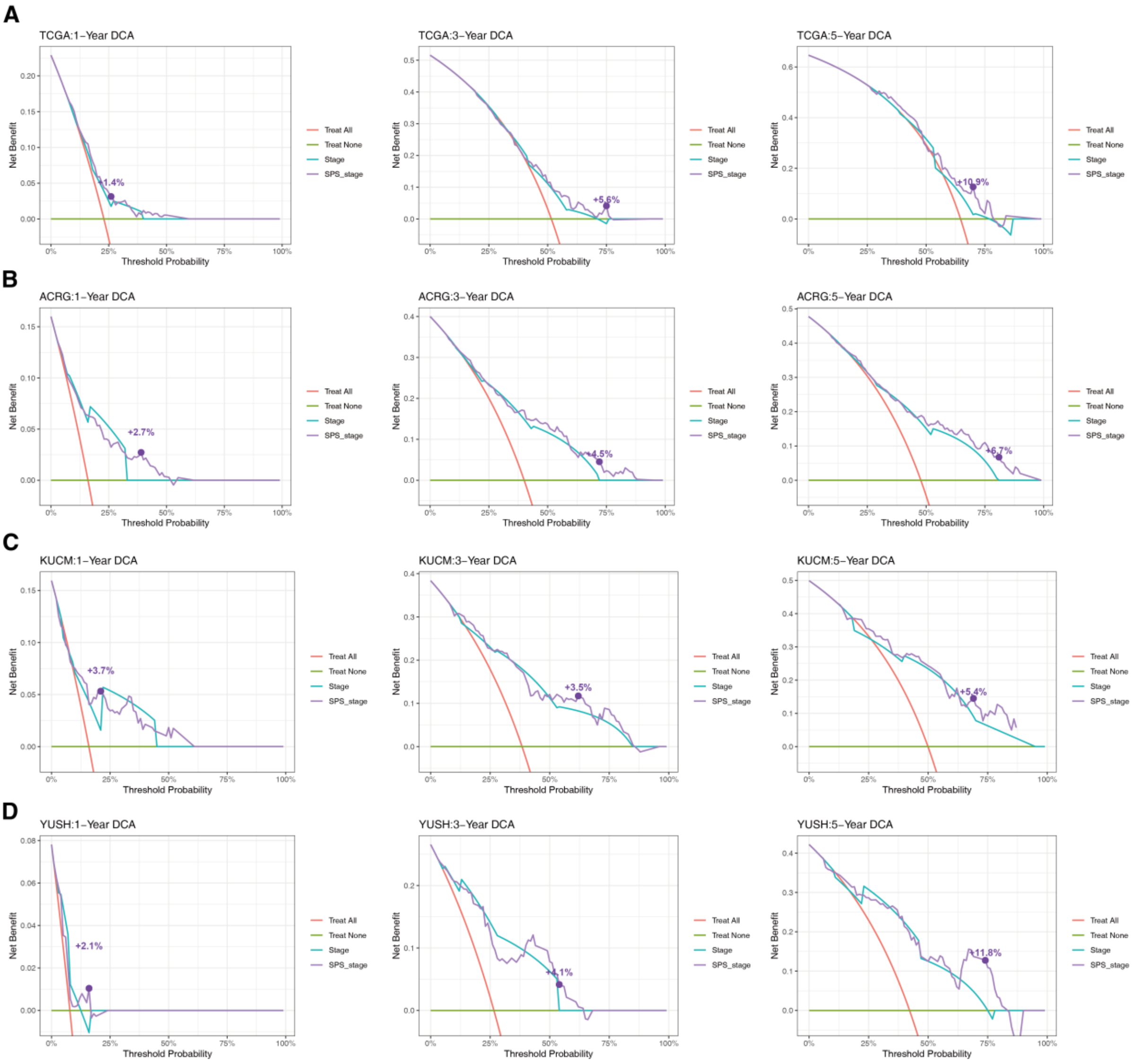

Figure S8. Decision curve analysis (DCA) comparing the SPS_stage and Stage models for predicting 1-, 3-, and 5-year overall survival (OS) in STAD.

Decision curves for TCGA **(A)**, ACRG **(B)**, KUCM **(C)** and YUSH **(D)** datasets.

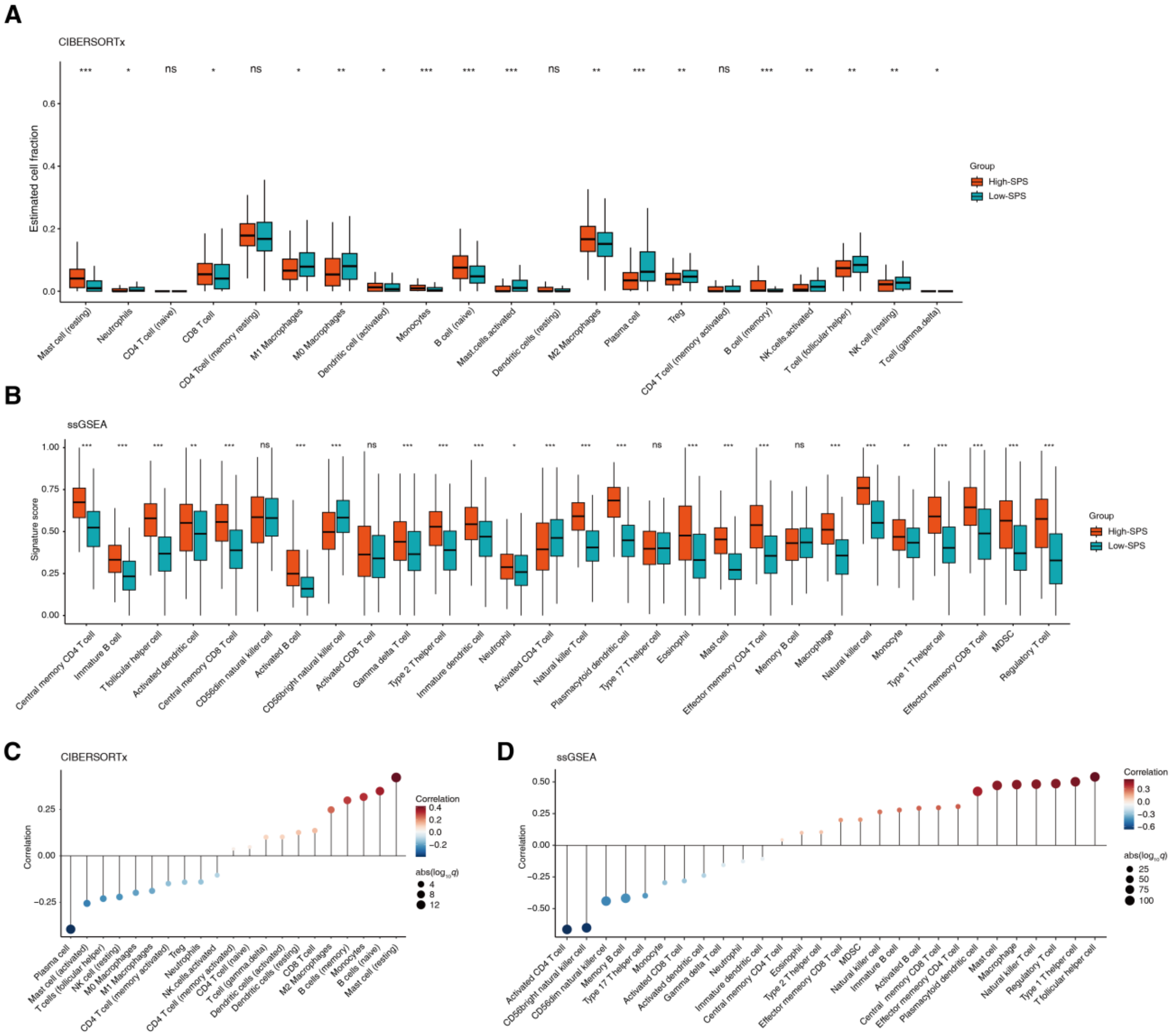

Figure S9. Differences in immune infiltration characteristics in the high- and low-SPS groups.

(A-B) Differential immune cell infiltration in high- and low-SPS groups using CIBERSORTx **(A)** and ssGSEA **(B)**. **(C-D)** Lollipop plot of correlation of SPS with immune cells using CIBERSORT **(C)** and ssGSEA **(D)**. Correlations were assessed using Spearman’s rank correlation. Group comparisons were performed using two-sided Wilcoxon rank-sum test. *$P < 0.05$, **$P < 0.01$, ***$P < 0.001$.

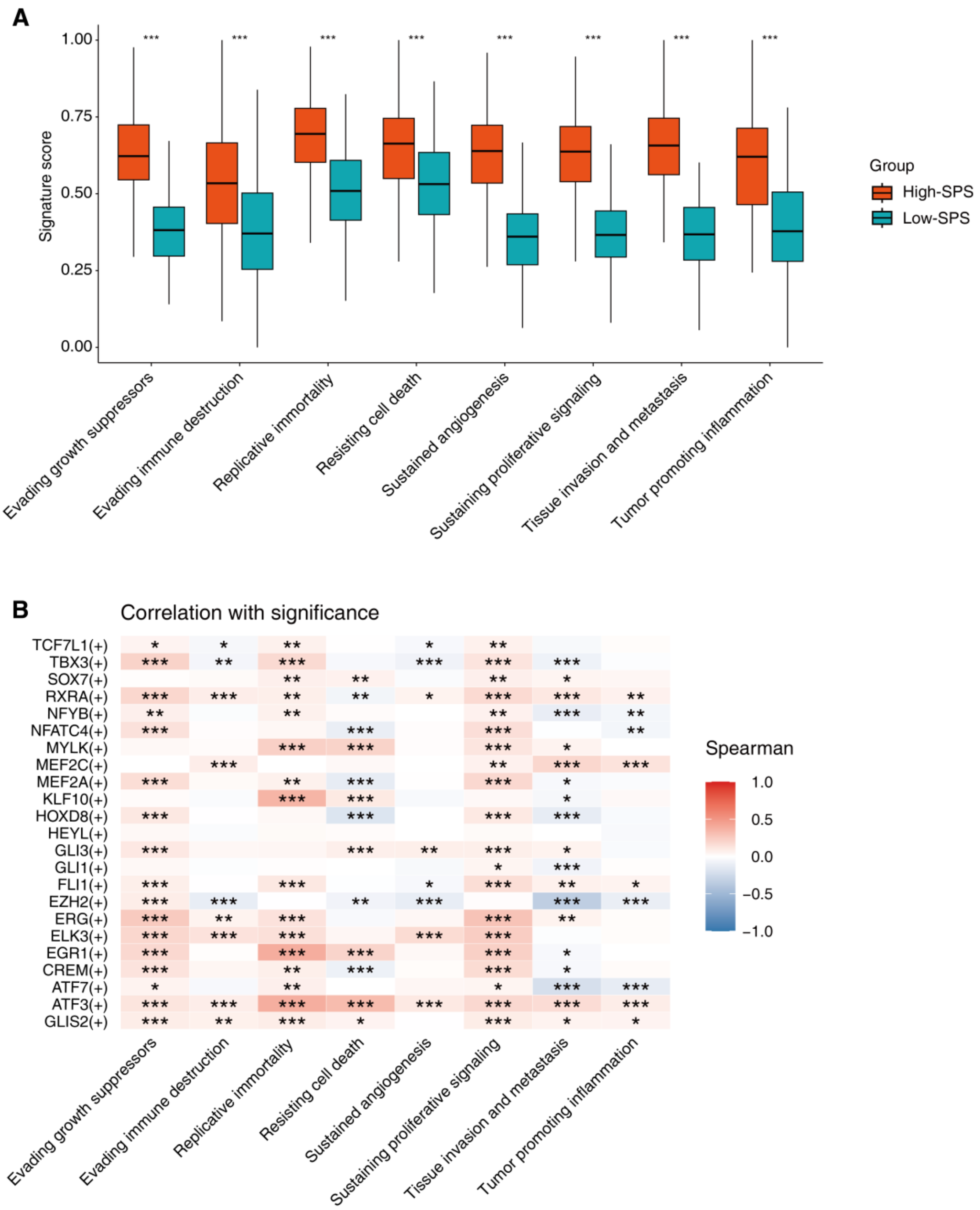

Figure S10. Functional characteristics of the SPS regarding cancer hallmarks.

(A) Differential cancer hallmark characteristics in high- and low-SPS groups using ssGSEA. **(B)** Heatmap illustrating the correlation between the activity of the 23 transcriptional regulators of SPS and cancer hallmark pathways. The color gradient represents the strength and direction of the association. Correlations were assessed using Spearman's rank correlation. Group comparisons were performed using two-sided Wilcoxon rank-sum test. *$P < 0.05$, **$P < 0.01$, ***$P < 0.001$.

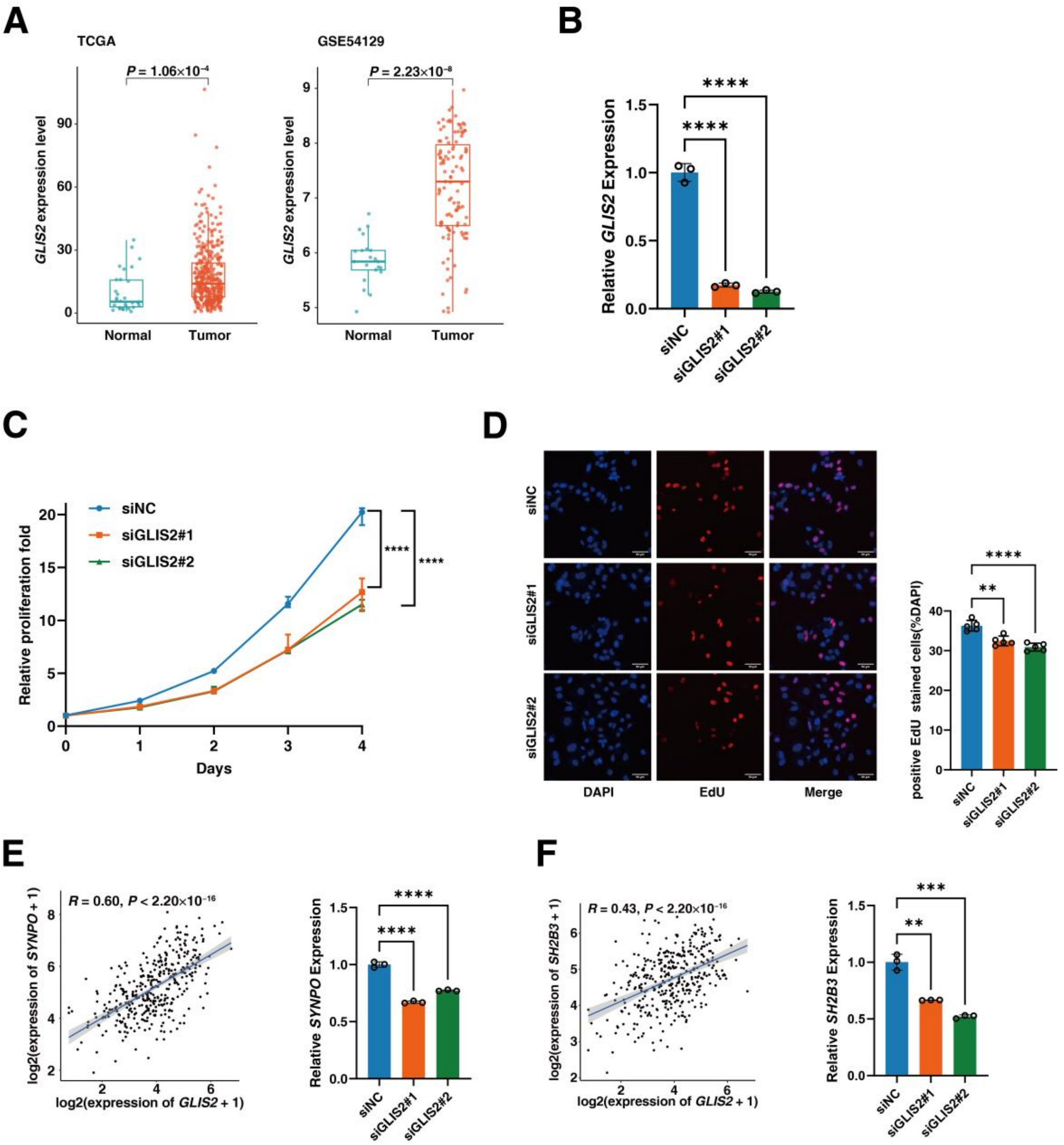

Figure S11. ***GLIS2*** **knockdown suppresses gastric cancer cell proliferation.**

(A) *GLIS2* was significantly overexpressed in tumor tissues compared with normal tissues in TCGA and GSE54129 datasets. Statistical significance was determined by Wilcoxon rank-sum test. **(B)** RT-qPCR analysis of *GLIS2* mRNA levels in AGS cells following *GLIS2* knockdown (n = 3). Statistical significance was assessed using a one-way ANOVA followed by Dunnett's multiple comparisons test. **(C)** Proliferation of AGS cells was evaluated by CCK-8 assays after *GLIS2* knockdown (n = 5). Statistical significance was assessed by a two-way ANOVA followed by Dunnett's multiple comparisons test. **(D)** Representative images and quantification of EdU assays in AGS cells after *GLIS2* knockdown (n = 5). Scale bar, 50 μm. Statistical significance was

assessed using a one-way ANOVA followed by Dunnett's multiple comparisons test. **(E-F)** Left: Correlation analysis of *GLIS2* expression with *SYNPO* **(E)**, or *SH2B3* **(F)** in TCGA dataset (Spearman's correlation). Right: RT-qPCR analysis of *SYNPO* **(E)** and *SH2B3* **(F)** mRNA levels after *GLIS2* knockdown in AGS cells (n = 3). Statistical significance was assessed using a one-way ANOVA followed by Dunnett's multiple comparisons test. All these Data are represented as mean ± SD. ** $P < 0.01$, *** $P < 0.001$, **** $P < 0.0001$.

Table S2. Comparison of C-index values: SPS_ stage model *vs.* Stage model.

Datasets	$\Delta_{C\text{-index}}$[a]	95% CI[b]	*P* value[b]
TCGA (n=376)	0.019	0.004–0.035	0.007
ACRG (n=300)	0.014	0.001–0.025	0.014
KUCM (n=109)	0.016	-0.004–0.036	0.059
YUSH (n=64)	0.017	-0.015–0.049	0.136

[a]$\Delta_{C\text{-index}}$: C-index (SPS_stage)-C-index (Stage);

[b]Confidence intervals and *P* values for $\Delta_{C\text{-index}}$ were calculated using 1,000 bootstrap resamples.

Table S3. Datasets used in this study.

Data Types	Datasets	Number of STAD patients
scRNA	GSE134520	38525 cells from 9 patients
Bulk RNA	TCGA	376
microarray datasets	GSE62254 (ACRG)	300
	GSE26901 (KUCM)	109
	GSE13861 (YUSH)	64

Table S4. siRNA sequences.

siRNA	sequences
siCLIS2 #1	GUCAACGAUUACCAUGUCATT
	UGACAUGGUAAUCGUUGACTT
siCLIS2 #2	CUCUCAAUCUGGCCAAGAATT
	UUCUUGGCCAGAUUGAGAGTT

Table S5. RT-qPCR primer sequences.

Gene Symbol	Direction	Sequences（5' to 3'）
GAPDH	Forward	GGAGCGAGATCCCTCCAAAAT
	Reverse	GGCTGTTGTCATACTTCTCATGG
CLIS2	Forward	CAGCCACTGCGCTATTTGGA
	Reverse	ACTTGTCCTTGGGAGGGGTAA
SYNPO	Forward	GCCCAACTCCCATCTAATGGC
	Reverse	GGGATGAGCGTAGCTTCTCTG
SH2B3	Forward	GTGGGGAATACGTGCTCACTT
	Reverse	TGTCCACGACCGAGGGAAA
